# Supplementary figures and images for: Inhibition of extracellular signal-regulated kinase pathway suppresses tracheal stenosis in a novel mouse model
Source: PLoS One. 2021 Sep 29;16(9):e0256127. doi: 10.1371/journal.pone.0256127 (PMC8480895; doi:10.1371/journal.pone.0256127)

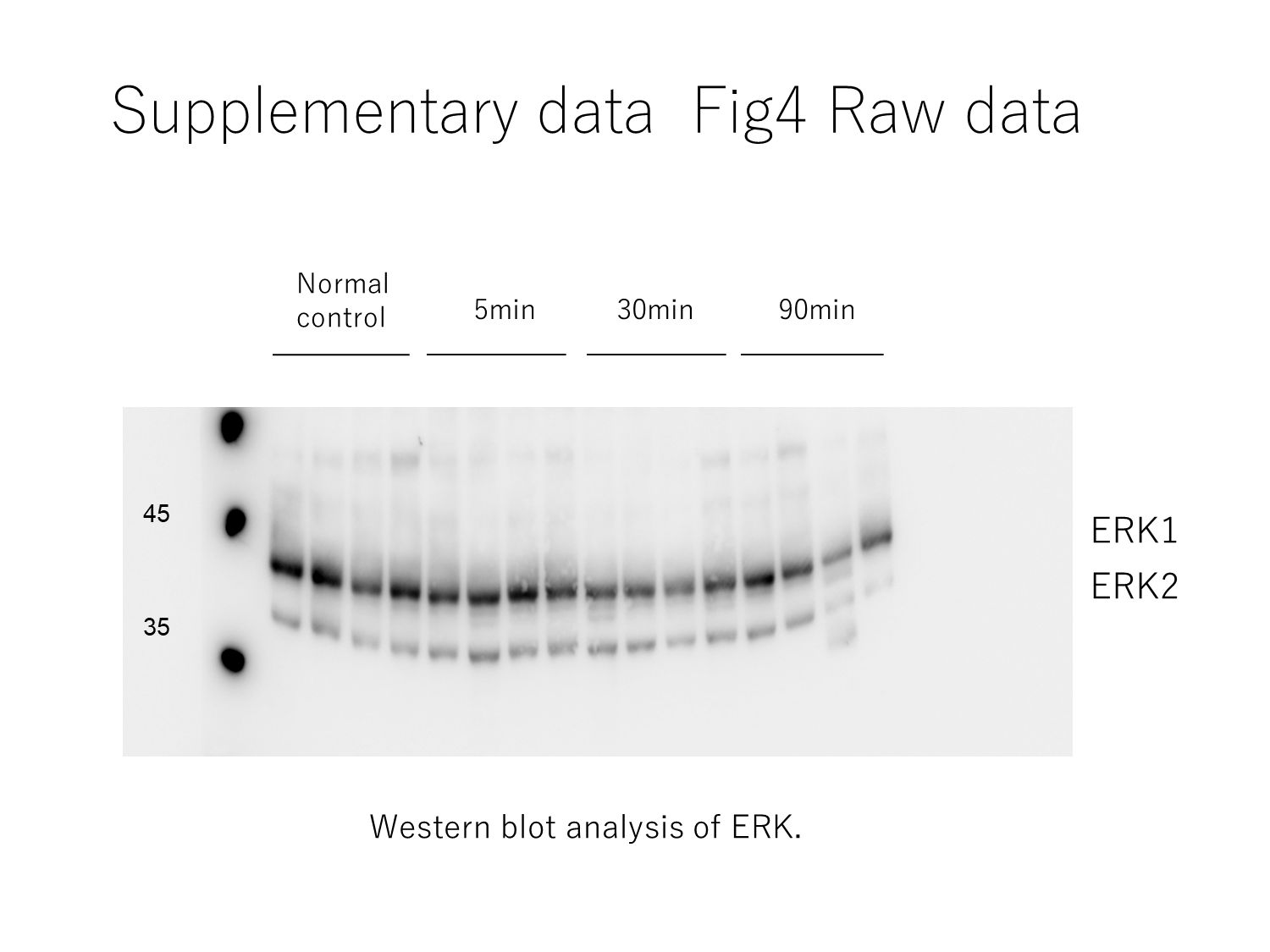

Supplement: S1 Fig — Western blot analysis of ERK and its associated β-actin loading control. (TIF) [file pone.0256127.s001.TIF]

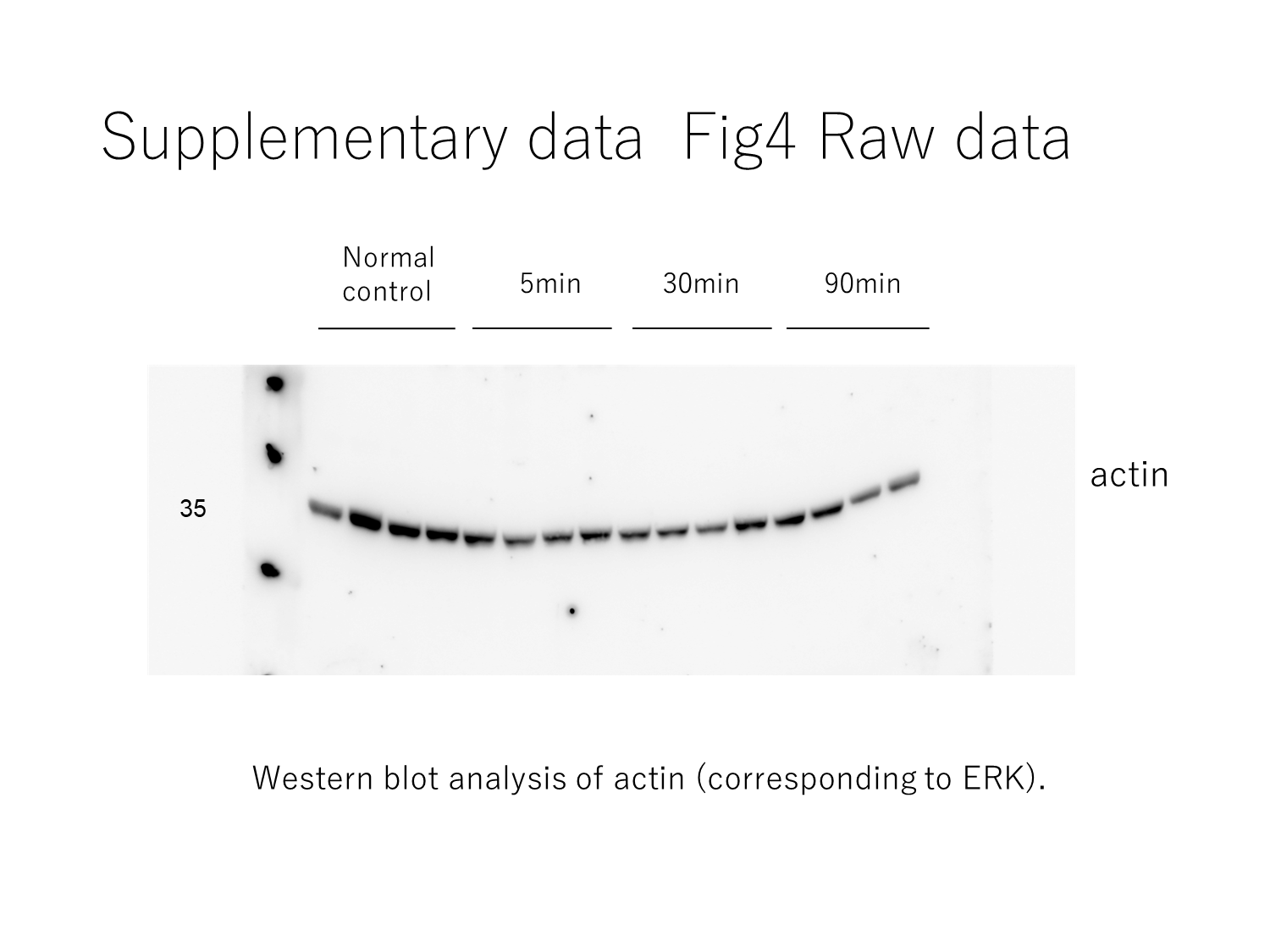

Supplement: S2 Fig — Western blot analysis of ERK and its associated β-actin loading control. (TIF) [file pone.0256127.s002.TIF]

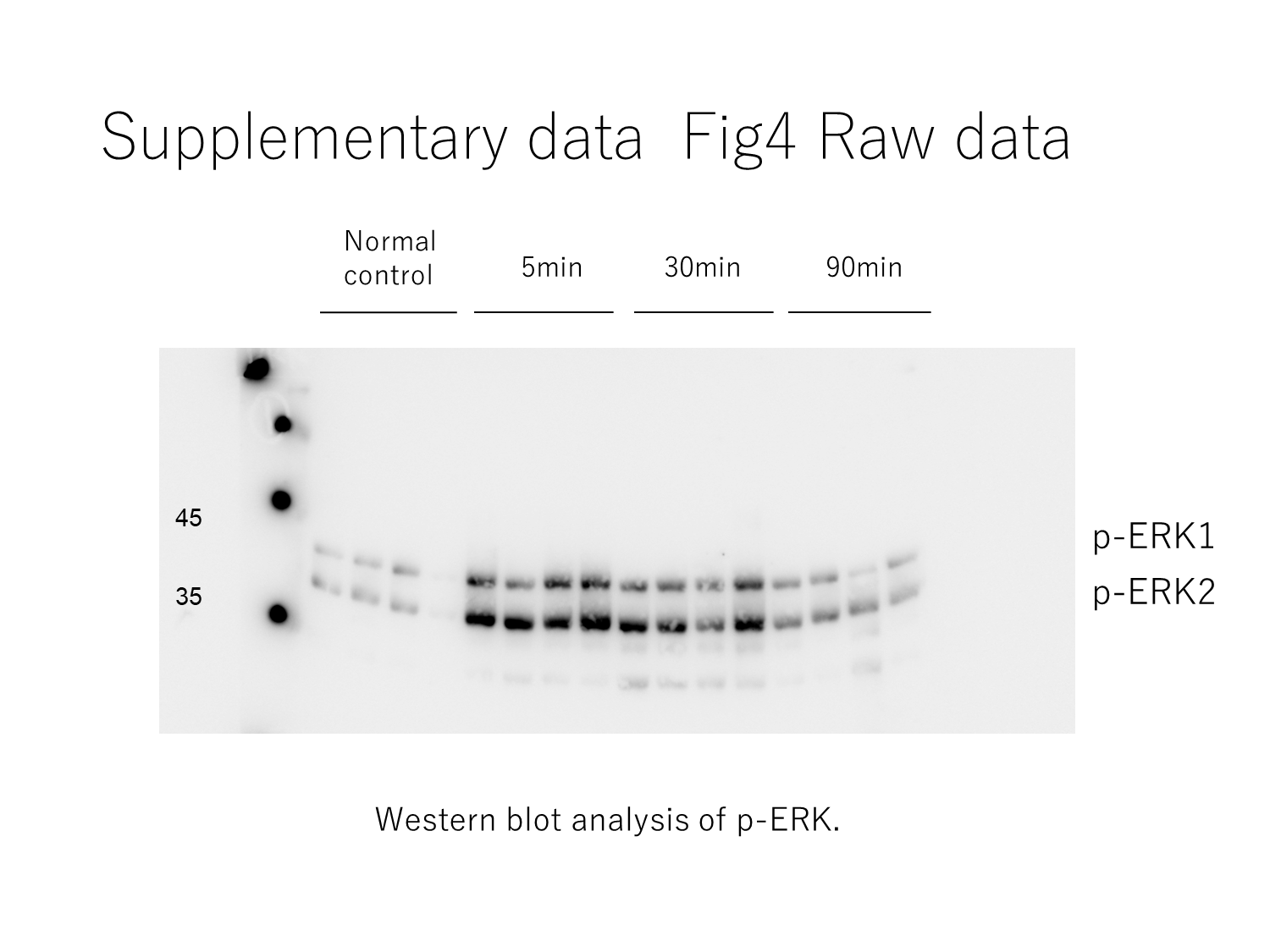

Supplement: S3 Fig — Western blot analysis of p-ERK and its associated β-actin loading control. (TIF) [file pone.0256127.s003.TIF]

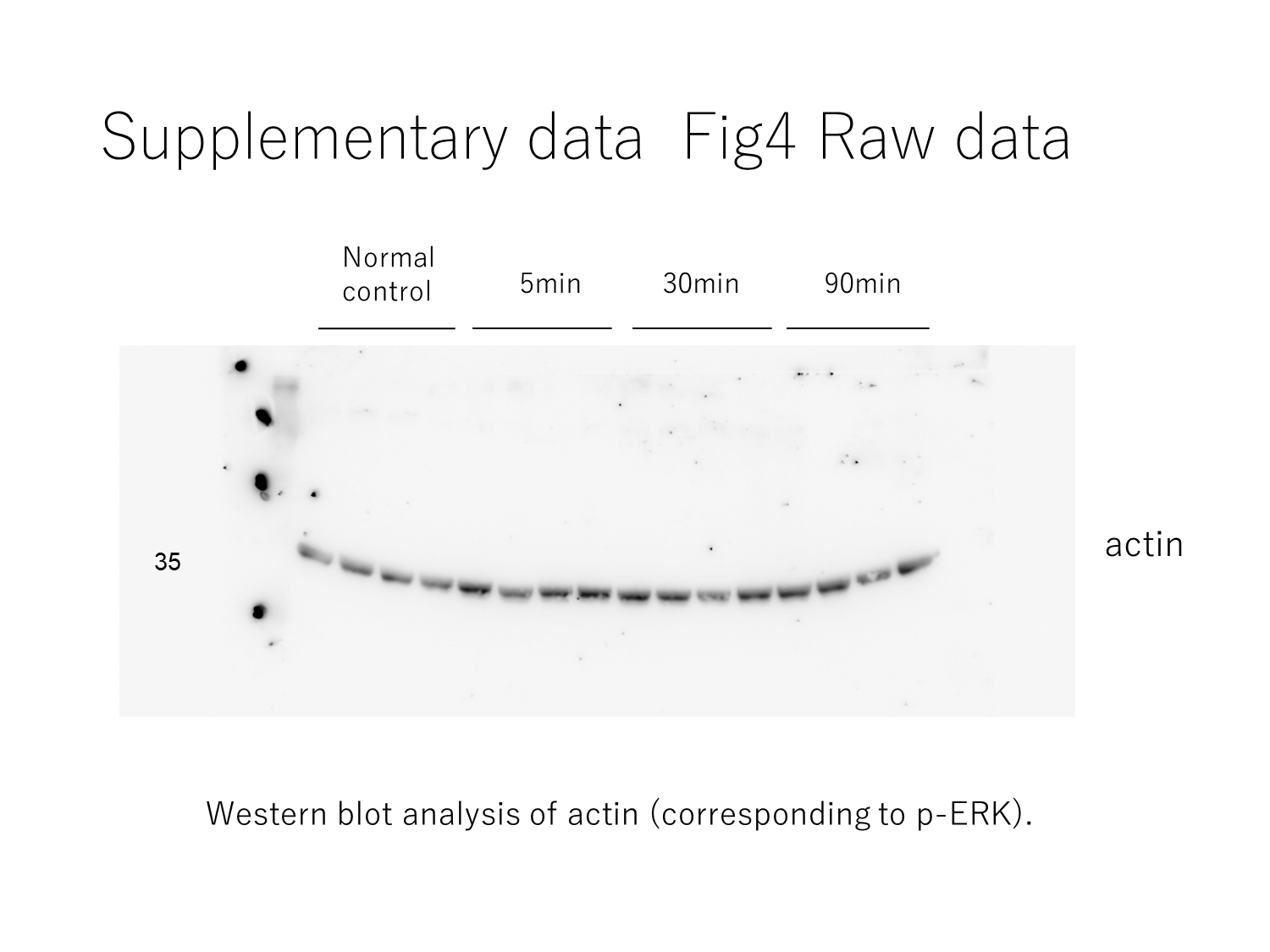

Supplement: S4 Fig — Western blot analysis of p-ERK and its associated β-actin loading control. (TIF) [file pone.0256127.s004.TIF]

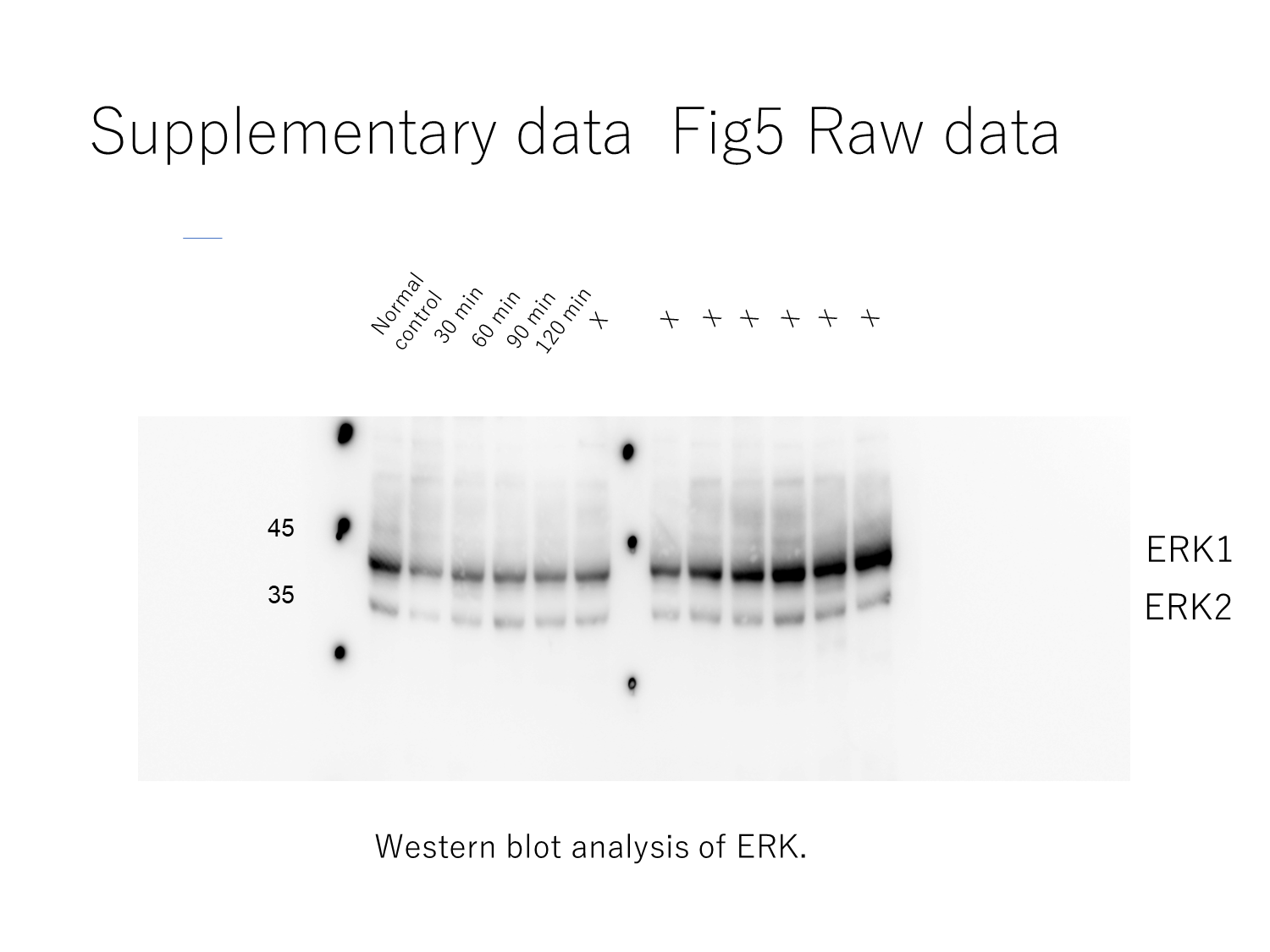

Supplement: S5 Fig — Western blot analysis of ERK and its associated β-actin loading control. (TIF) [file pone.0256127.s005.TIF]

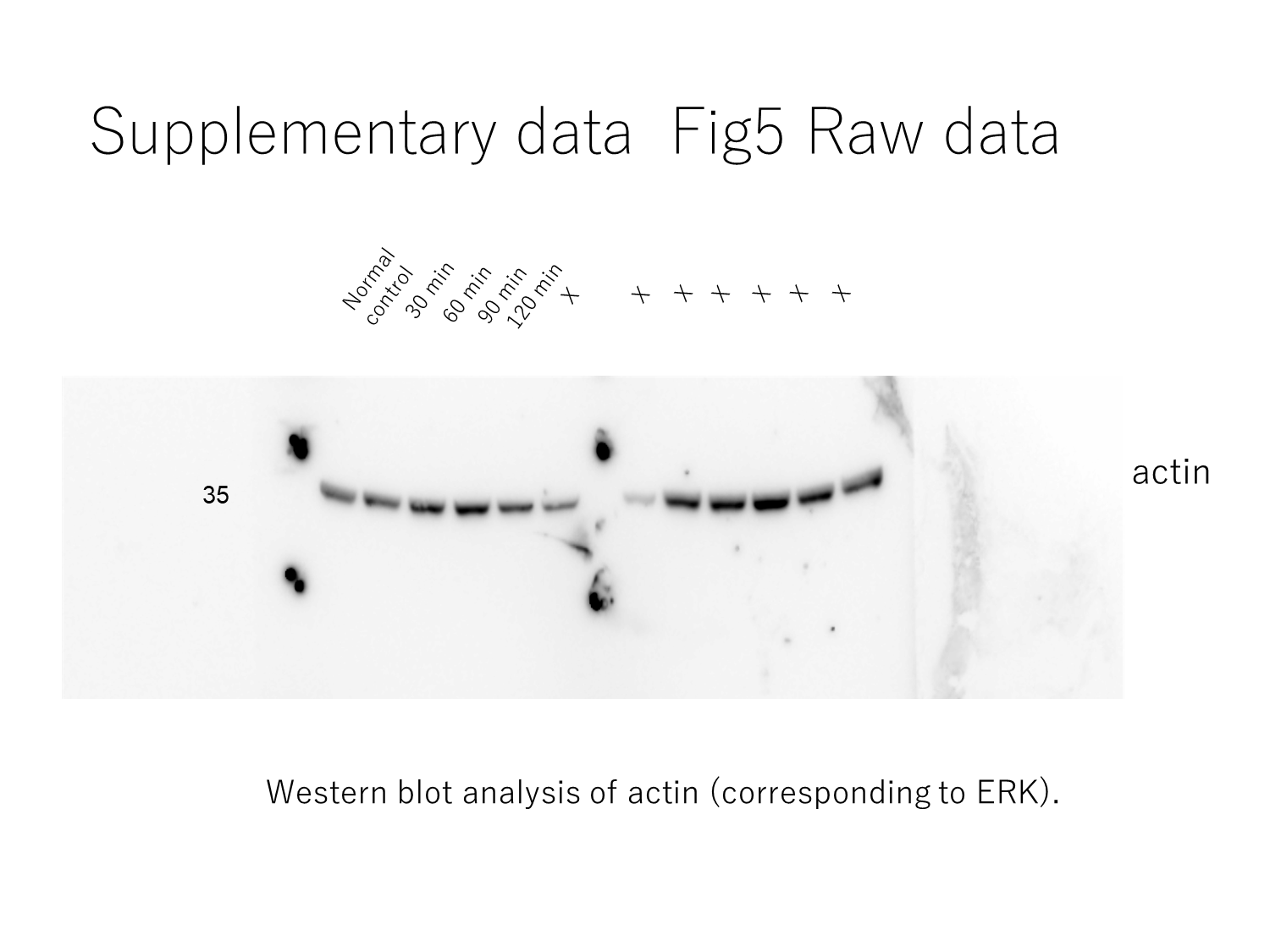

Supplement: S6 Fig — Western blot analysis of ERK and its associated β-actin loading control. (TIF) [file pone.0256127.s006.TIF]

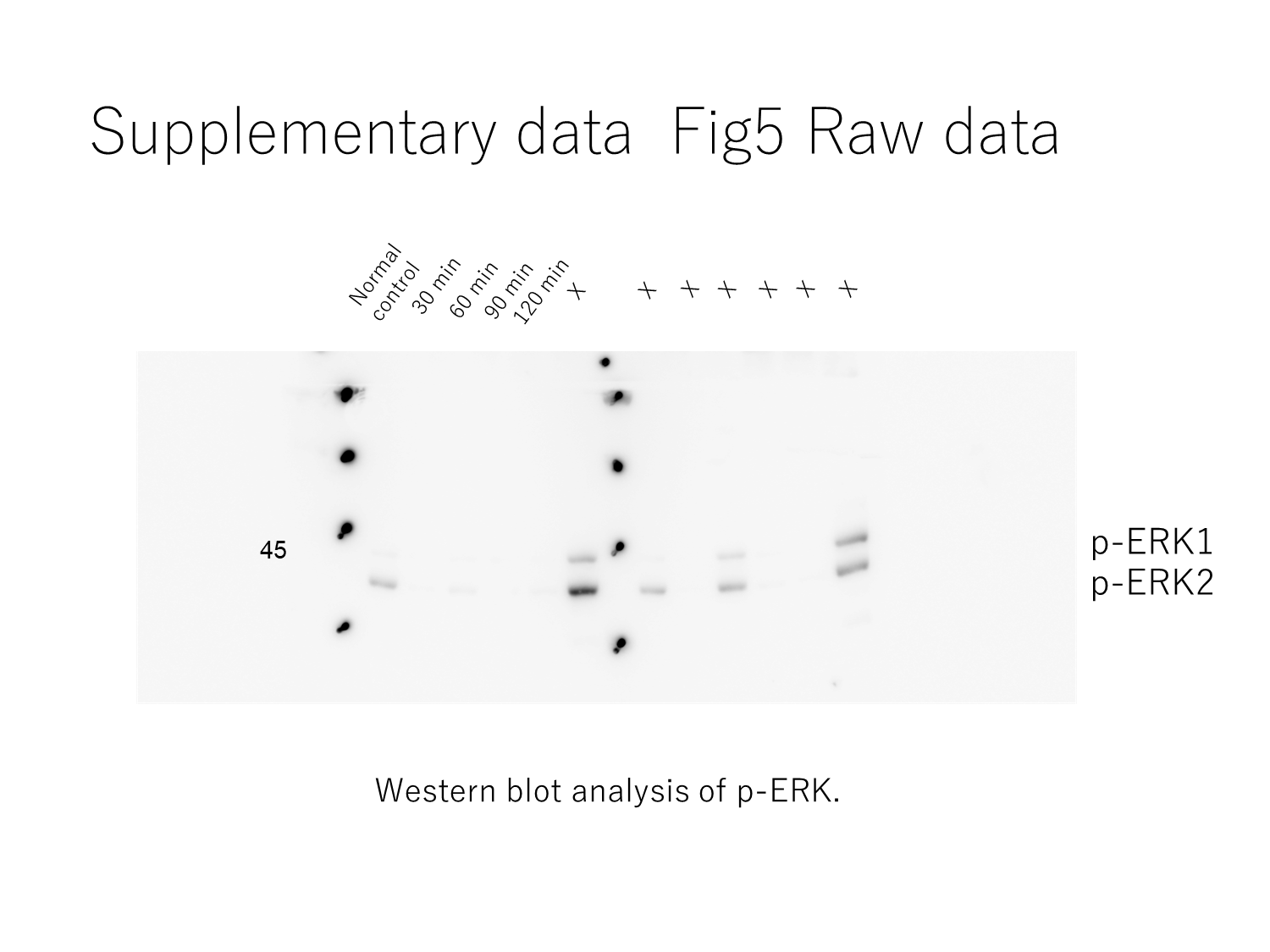

Supplement: S7 Fig — Western blot analysis of p-ERK and its associated β-actin loading control. (TIF) [file pone.0256127.s007.TIF]

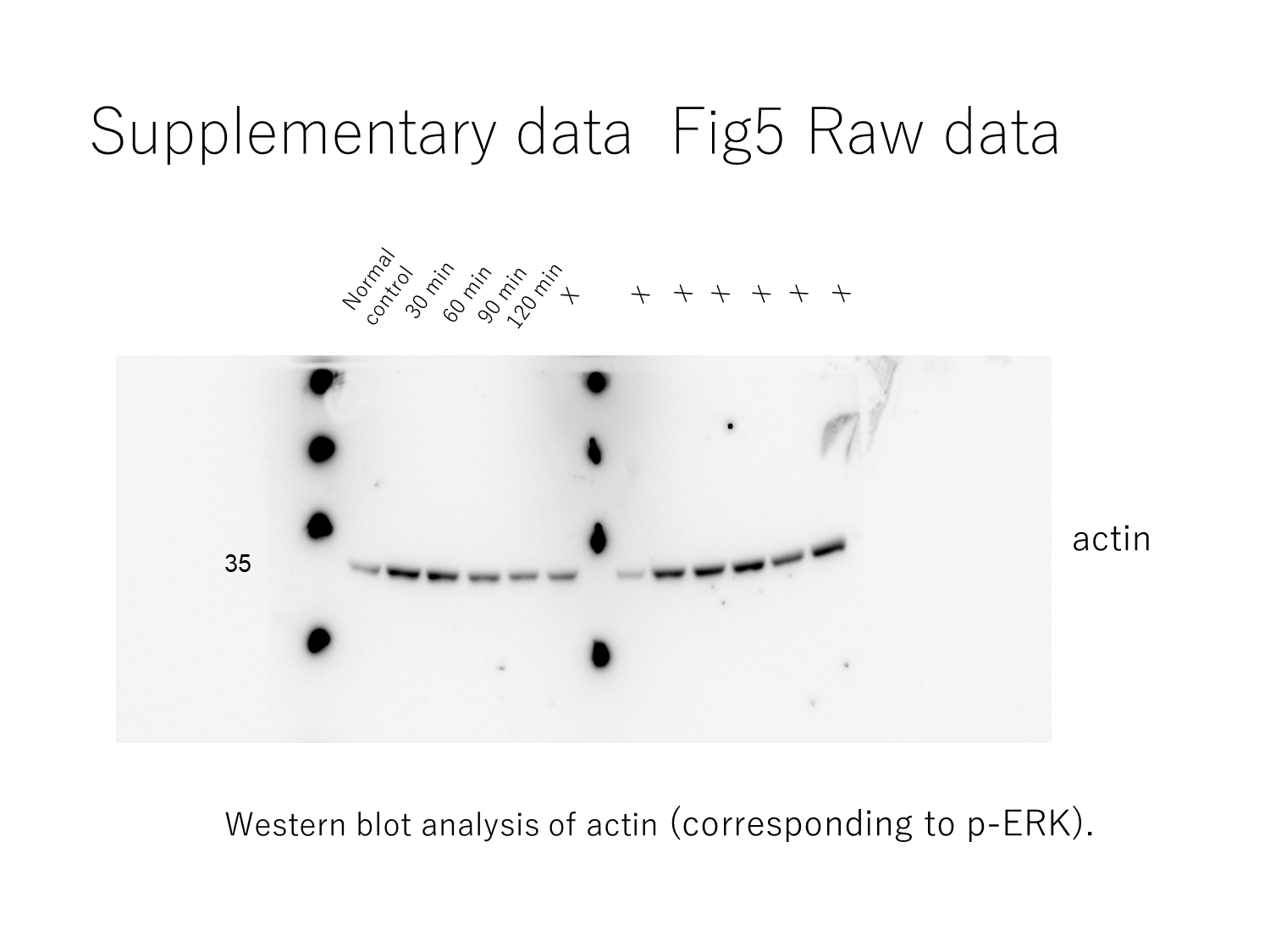

Supplement: S8 Fig — Western blot analysis of p-ERK and its associated β-actin loading control. (TIF) [file pone.0256127.s008.TIF]

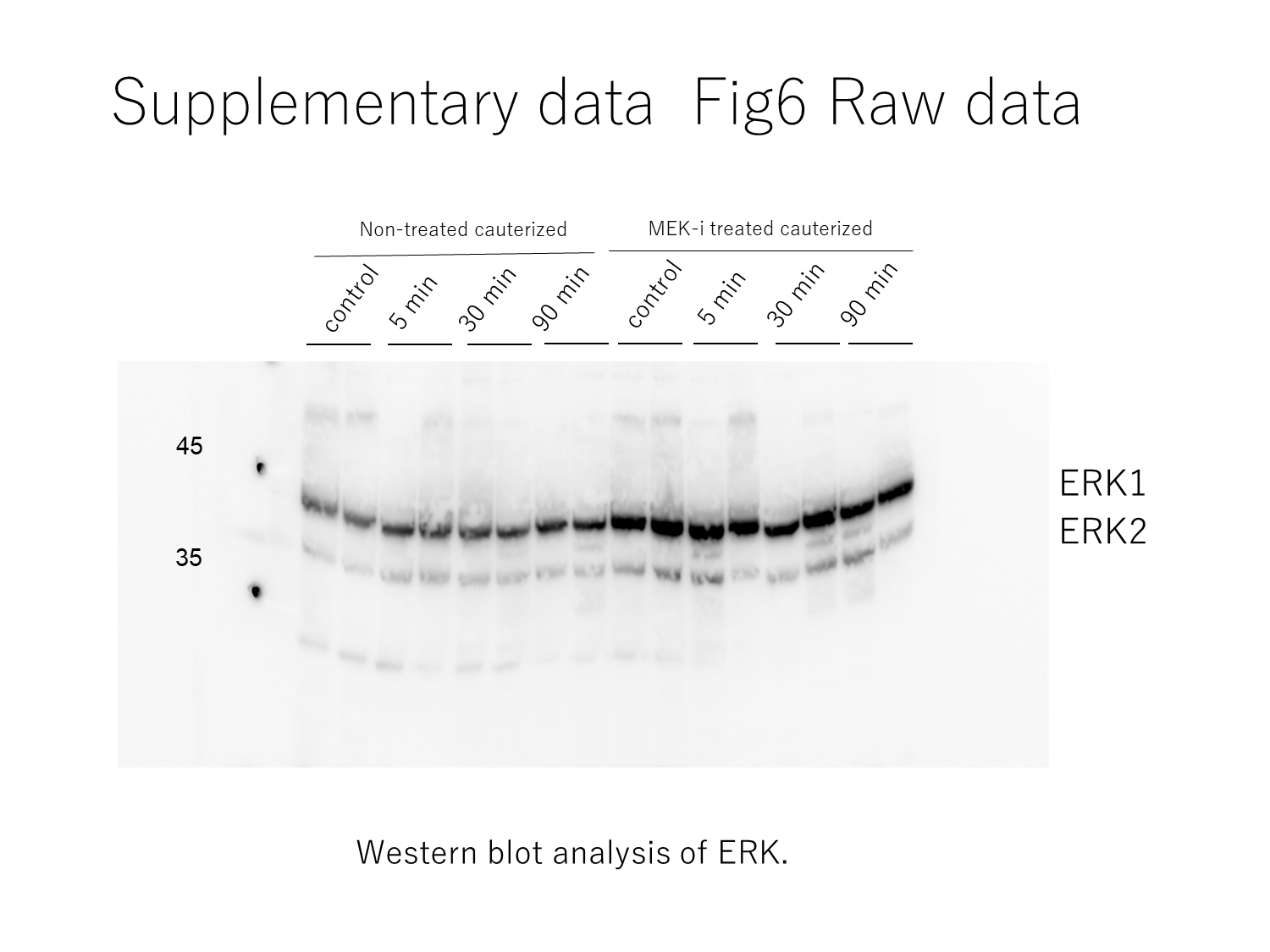

Supplement: S9 Fig — Western blot analysis of ERK and its associated β-actin loading control. (TIF) [file pone.0256127.s009.TIF]

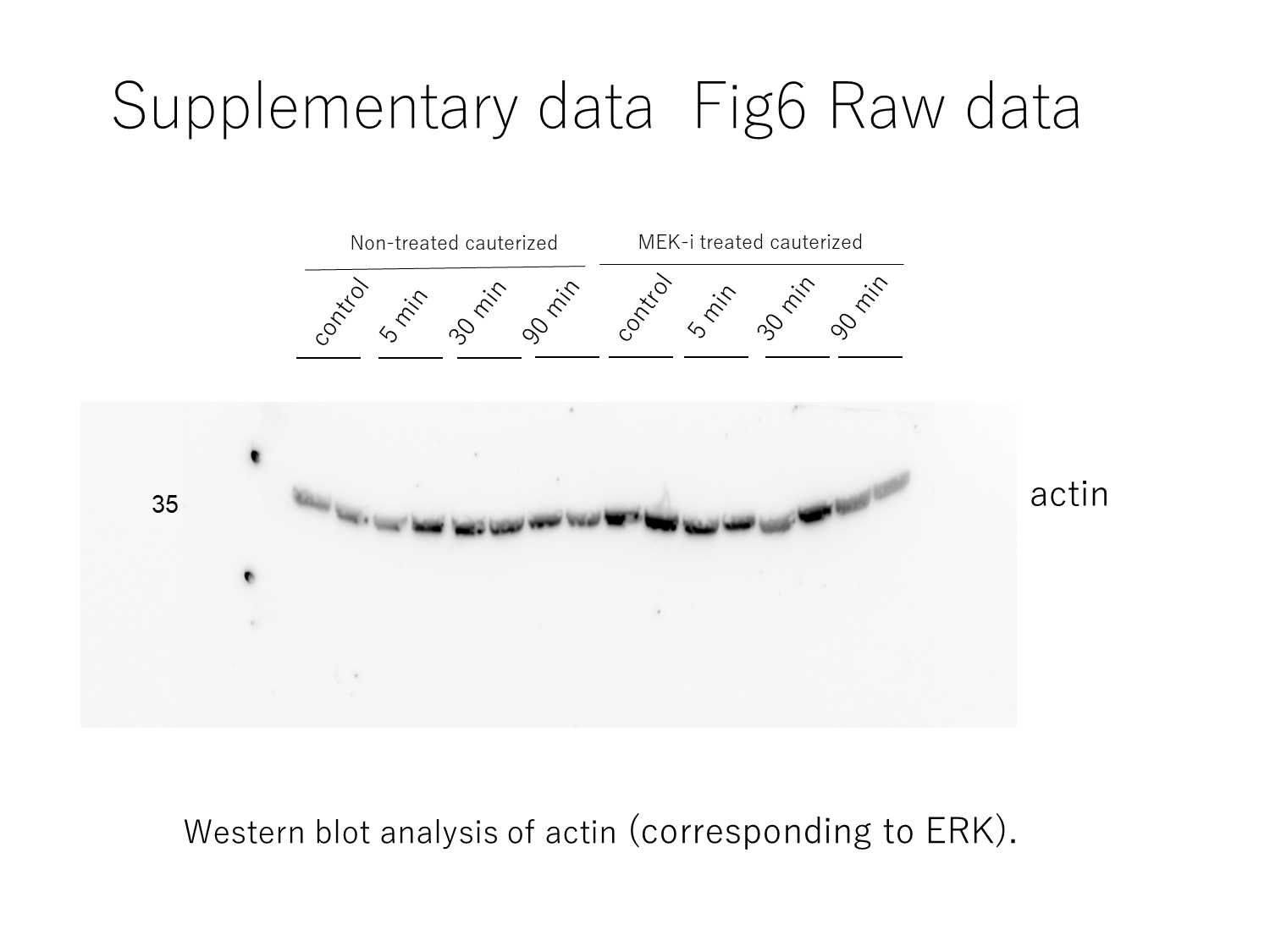

Supplement: S10 Fig — Western blot analysis of ERK and its associated β-actin loading control. (TIF) [file pone.0256127.s010.TIF]

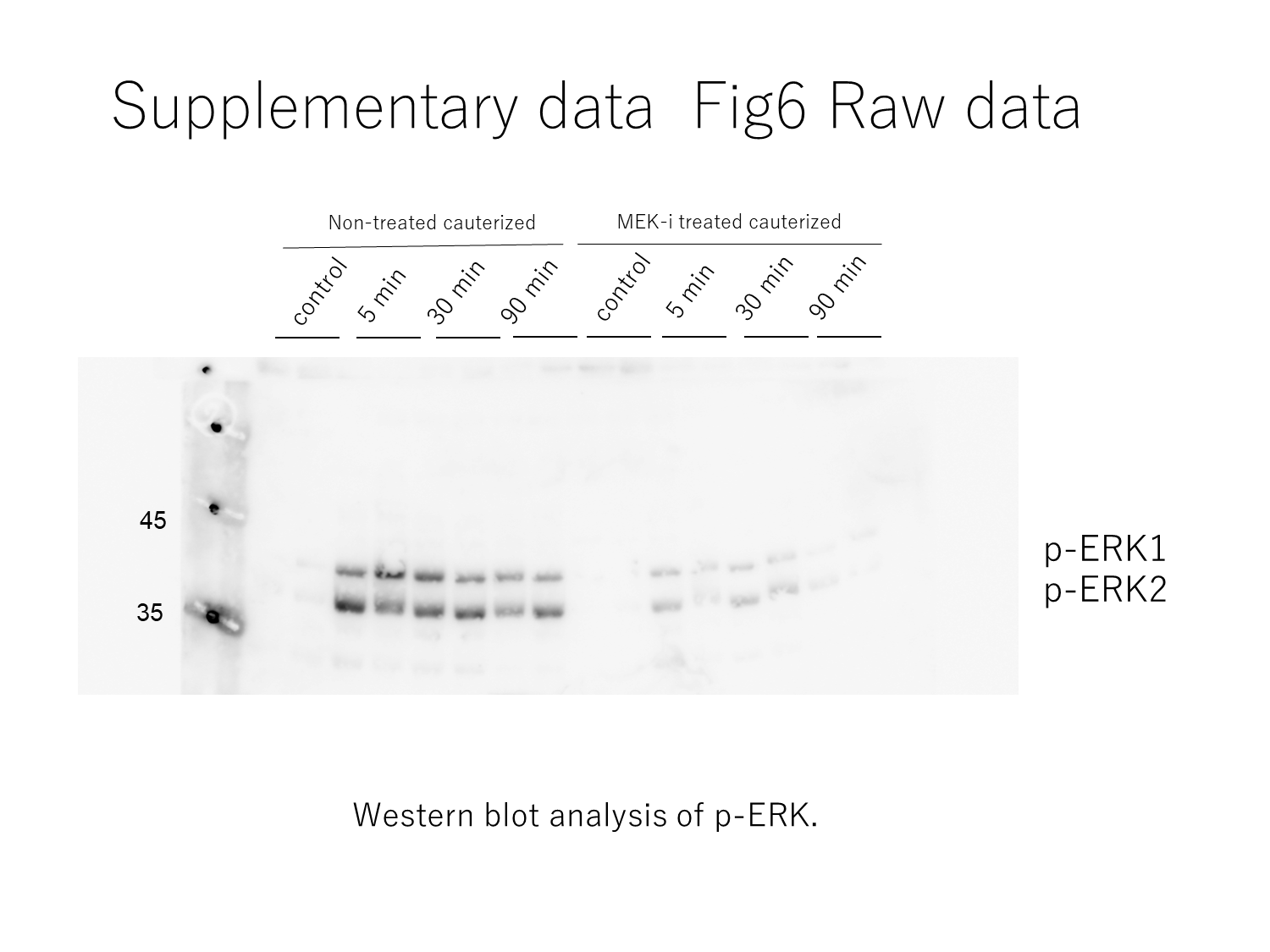

Supplement: S11 Fig — Western blot analysis of p-ERK and its associated β-actin loading control. (TIF) [file pone.0256127.s011.TIF]

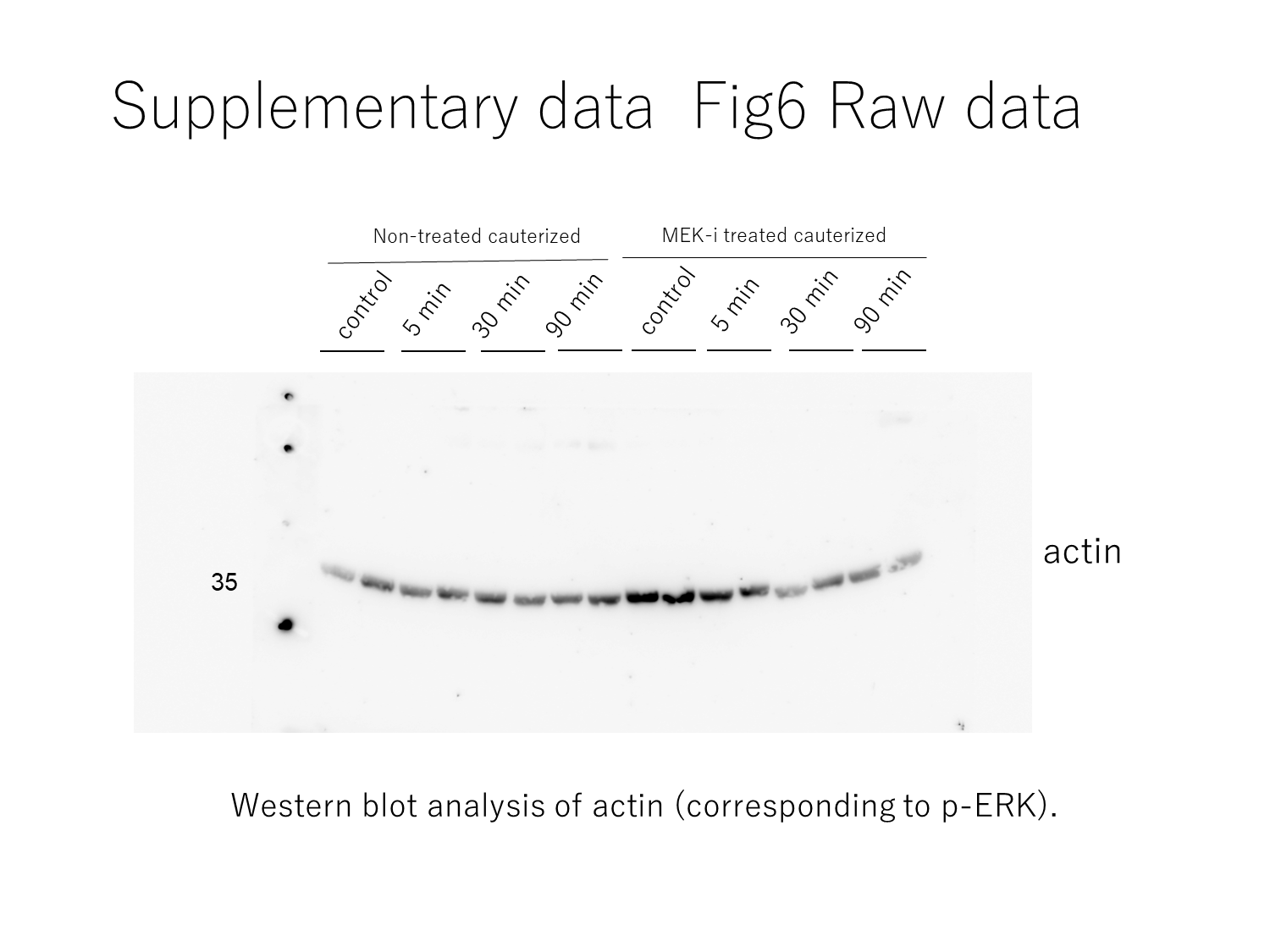

Supplement: S12 Fig — Western blot analysis of p-ERK and its associated β-actin loading control. (TIF) [file pone.0256127.s012.TIF]
